# Assessing the risk of low energy availability, bone mineral density and psychological strain in endurance athletes

**DOI:** 10.1080/15502783.2025.2496448

**Published:** 2025-04-22

**Authors:** Charlotte R. Gowers, Christopher J. McManus, Henry C. Chung, Ben Jones, Jamie Tallent, Sally P. Waterworth

**Affiliations:** a University of Essex, School of Sport, Rehabilitation, and Exercise Sciences, Colchester, UK; b Monash University, Department of Physiotherapy, Faculty of Medicine, Nursing and Health Sciences, School of Primary and Allied Health Care, Melbourne, Australia

**Keywords:** Low energy availability, LEA, LEAF-Q, bone mineral density, osteopenia

## Abstract

**Background:**

Adequate energy intake is crucial for athletic performance and recovery. However, many endurance athletes experience Low Energy Availability (LEA), which, if prolonged, can detrimentally impact both health and performance.

**Methods:**

A total of 55 endurance athletes (23 females; 45  ±  13 years, 1.64  ±  0.06 m, 64.4  ±  11.4 kg and 32 males; 44  ±  13 years, 1.76  ±  0.18 m, 78.8  ±  9.2 kg) underwent physical assessments and completed questionnaires on dietary habits, training loads, and psychological stress. Dual-Energy X-ray Absorptiometry (DEXA) scans measured bone mineral density (BMD) in the lumbar L1-L4 spine, and body composition. Risk of LEA burnout, and psychological strain were assessed using sport-specific questionnaires.

**Results:**

Seventy-seven percent of female athletes were identified as at risk of LEA by the LEAF-Q. These females had higher body weight and fat percentage than those at low risk of LEA. Male athletes had a higher prevalence of low lumbar BMD (31%) compared to females, associated with older age, and longer training histories. Although only 9% of female athletes had low-BMD, those affected had a history of amenorrhea and were identified as at risk of LEA by the LEAF-Q.

**Conclusion:**

A high proportion of endurance athletes had low-BMD and were at risk of LEA. This underscores the need for targeted nutritional strategies to mitigate the risks associated with LEA and promote overall athlete well-being.

## 1. Introduction

Endurance sports are characterized by prolonged durations of continuous or intermittent effort. The high energy demands and unique physiological adaptations associated with endurance training necessitate adequate energy intake for optimal performance, recovery, and overall health [1–3]. Low energy availability (LEA) occurs when energy intake does not meet energy requirements, resulting in insufficient energy to support normal physiological functions after accounting for exercise energy expenditure [4]. LEA can be unintentional (due to a lack of awareness of energy demands) or intentional (efforts to reduce body fat and/or weight), and can occur with or without an eating disorder (ED) or disordered eating behaviors [5].

Prolonged or recurrent LEA can lead to Relative Energy Deficiency in Sport (REDs), a condition encompassing a spectrum of health and performance impairments [6]. REDs is recognized as a significant concern for both male and female athletes. The prevalence of LEA/REDs varies considerably, ranging from 23% to 79.5% for females and 15% to 70% for males across different sports, with the highest rates observed in endurance athletes [6]. LEA impacts various physiological functions, leading to detrimental effects on health and performance over time. These effects include metabolic disturbances such as suppression of resting metabolic rate [7,8], menstrual dysfunction in female athletes [9–11], impaired bone health [12], compromised cardiovascular profiles [13,14], and psychological challenges [15,16]. Athletes experiencing high mechanical loading typically have higher bone mineral density (BMD) than age-matched non-athletes [17]. However, several studies have shown that endurance athletes, particularly long-distance runners, have lower BMD compared to non-athletes or athletes in contact sports [18], with the most pronounced differences observed at the lumbar spine [19]. The prevalence of low-BMD varies between studies, with incidences ranging from 13–50% for osteopenia (Z-score between  − 1 and  − 2) and 0–13% for osteoporosis (Z-score ≤-2) [20–22]. This is concerning due to the increased risk of acute stress fractures [2] and fragility fractures (low trauma fractures sustained from a fall from standing height or less) in later adulthood [23].

The effects of LEA can vary between athletes, and are influenced by the depth and duration of the energy deficit, as well as individual characteristics such as sex, age, and sport [5]. Female athletes often consume less energy and carbohydrates per kilogram of body weight compared to males [24], and are more adversely affected by reduced carbohydrate and energy availability [2]. Recent research, however, indicates an increasing prevalence of LEA among male endurance athletes [6]. In males, LEA can lead to reduced testosterone levels and libido, which are more challenging to identify compared to menstrual irregularities in females. The Low Energy Availability in Females Questionnaire (LEAF-Q) is a validated tool for identifying female athletes at risk of LEA [25]. Efforts have been made to develop a similar tool for males (LEAM-Q) but further validation is needed [26]. This indicates the infancy and lack of research within the area and the necessity to explore this in further detail, especially within men.

Prolonged LEA can also impact mental health, potentially leading to or exacerbating conditions such as disordered eating behaviors, depression, and anxiety [27,28]. The relationship between LEA and psychological factors is complex and multifactorial, with psychological stress possibly triggering LEA and, conversely, LEA contributing to psychological distress. Athletes often face a multifaceted mix of pressures from rigorous training schedules, professional commitments, and personal life demands. This cumulative stress, driven by the intense desire to achieve and excel, can significantly elevate stress levels. Over time, sustained high stress may lead to more severe conditions, such as Burnout Syndrome in Athletes and other mental health disturbances [29]. This is concerning for mental well-being and can also diminish athletic performance, increase the risk of injury, and lead to a disengagement from the sport [30]. Furthermore, athletes struggling with mental health issues may experience a reduction in motivation, mood changes, and a decrease in performance, which could perpetuate the cycle of stress and LEA. Recognizing and addressing these issues early is important for the long-term health and career sustainability of athletes [29].

Despite the growing body of evidence highlighting the detrimental effects of LEA in both men and woman, our understanding remains incomplete. Much of the available research has been conducted in elite athletes and there is a need to further investigate the implications of LEA on bone health in non-elite athletes, and explore the interactions between physiological and psychological aspects of LEA. The aims of this study were to investigate the prevalence of LEA and low lumbar BMD in an endurance athlete population and explore the association with anthropometric (e.g. fat mass, lean mass), cardiovascular (e.g. blood pressure), metabolic (e.g. glucose levels, lipid profile) and psychological measures (e.g. stress, burnout).

## 2. Methods

### 2.1. Participants

Participants were recruited from local clubs, athlete forums, and university performance centers via e-mails and social media posts. Fifty-five endurance athletes (23 females 45  ±  13 years, 1.64  ±  0.06 m, 64.4  ±  11.4 kg, BMI 23.9  ±  3.8 kg/m^2^; 32 males 44  ±  13 years, 1.76  ±  0.18 m, 78.8  ±  9.2 kg, BMI 27.7  ±  17.4 kg/m^2^) were recruited from the UK East Anglia region and were all classed as Caucasian European origin with a British ethnicity. To be eligible for the study, participants were required to be ≥18 years, train for endurance sport (cycling, running, triathlon) at least three times per week for more than one year, and be free from injury and illness. Participants were excluded if they were pregnant, had a current or past diagnosis of an eating disorder, currently or previously smoked, had any medical conditions, or were taking any prescribed medications. A total of 66 participants expressed an interest in the study. All satisfied the inclusion and exclusion criteria; however 11 chose not to participate, primarily due to other commitments.

All participants gave written and informed consent and ethical approval was granted by the University of Essex ethics committee (ETH2122–1025).

### 2.2. Experimental design

In a cross-sectional study design, participants took part in one testing session. Participants presented to the research laboratory fasted (self-reported; no food, drink, or caffeine in the previous 5 hours) and having refrained from strenuous exercise and alcohol for at least 24-hours. Data were collected by the same researcher (CG) to ensure standardization of measures and procedures.

### 2.3. Anthropometric measures

Participants’ height and body mass were recorded using a stadiometer and electronic scales (Seca 213 and 813, Seca, Hamburg, Germany), with participants attired in minimal clothing. Waist circumference was measured at the narrowest part above the navel using an ergonomic circumference measuring tape (Seca 201, Seca, Hamburg, Germany).

### 2.4. Blood pressure

An automatic blood pressure monitor (OMRON Intellisense, OMRON Healthcare, Kyoto, Japan) was used to measure the participants’ resting blood pressure. An appropriately sized inflatable cuff was fitted around the upper left arm and participants were instructed to sit still in their chair with feet flat on the floor, and legs uncrossed and left forearm resting on a table with the palm facing upwards. A minimum of two measurements were obtained for both systolic (SBP) and diastolic (DBP) blood pressure and the lowest value was recorded. In the case of measurement error or when there was poor agreement between measures (difference of >20 mmHg SBP or >10 mmHg DBP), a third measurement was made.

### 2.5. Lipid profile

A 40 μL sample of capillary blood was drawn from the fingertip to evaluate the lipid profile (Cholestech LDX™, Abbott Laboratories, Chicago, USA). Measures of total cholesterol (TC; mmol/L), high-density lipoprotein (HDL; mmol/L) cholesterol, triglycerides (TRG; mmol/L), and blood glucose (GLU; mmol/L). The Cholestech LDX™ has previously demonstrated good agreement with laboratory measures for population-based risk factor screening and meets the criteria set by the lipid standardization panel for accuracy and precision of cholesterol measurements [31].

### 2.6. Body composition and bone health

Participants underwent two Dual Energy X-ray Absorptiometry (DEXA) scans using a pencil beam DEXA scanner (Hologic Discovery W, Hologic Inc., Marlborough, USA). This was calibrated daily and stable on daily phantom quality assessment (coefficients of variation = 0.22%). The scan order was standardized with the whole-body scan performed first, followed by the lumbar scan. For the whole-body scan, participants were positioned supine along the mid-line of the DEXA table, arms by their side and palms facing down, legs shoulder width apart and internally rotated, and feet taped together at the metatarsophalangeal joint to maintain a fixed position throughout the duration of the scan [32]. Whole-body scans were used to measure whole body fat mass (kg), lean mass (kg) and visceral fat mass (g). For the subsequent anterior-posterior lumbar spine (AP lumbar spine L1-L4) scan, participants were positioned in a supine position with a box placed under the popliteal crease of both knees to achieve a ∼90° angle. Scans were analyzed using associated software (APEX 2.3.1, Hologic Inc., Marlborough, USA) by the same technician for consistency. Lumbar scans were used to measure bone mineral density (BMD; g/cm^3^), bone mineral content (BMC; g), Z-score (matched for age, gender, and ethnicity), and T-score (compared with an average healthy 30-year-old adult). A Z-score of <-1 was considered an indication of osteopenia and was used for classification of low-BMD [33].

### 2.7. Questionnaires

Questionnaires were administered via an online platform (Qualtrics, Provo, USA) prior to physical testing. In addition to questions about age, sport, performance level, training hours, dietary and sleeping habits, and perceived pressure in sport, participants completed the LEAF-Q (females only), Athlete Burnout Questionnaire (ABQ), and Athlete Psychological Strain Questionnaire (APSQ).

The 25-item LEAF-Q was developed to identify female athletes at risk of LEA by utilizing subsets of gastrointestinal symptoms, injury frequency, and menstrual dysfunction [25]. The internal consistency of the LEAF-Q was confirmed with a Cronbach’s alpha of 0.75, 0.79 and 0.61 for gastrointestinal symptoms, injury frequency and menstrual dysfunction respectively, and test-retest reliability showed stability over a 2-week interval (ICC = 0.79) [25]. The 15-item ABQ consists of three subscales: reduced sense of accomplishment (RA; five items), emotional/physical exhaustion (E; five items), and devaluation (D; five items). A 5-point Likert scale ranging from 1 (almost never) to 5 (almost always) was used in accordance with the original ABQ [34]. The ABQ has demonstrated strong reliability with Cronbach’s alpha values greater than 0.86 [35], and its utility as a valid and reliable measure of athlete burnout has been confirmed by several studies [36,37]. The 10-item APSQ was used to investigate mental health within the athlete population, and provide cutoff scores to discriminate between moderate, “high,” and “very high” levels of psychological stress in athletes [38]. Internal consistency values for the APSQ have been shown to be in the acceptable range, and a series of receiver operating characteristic (ROC) curve analyses values for the three cutoff scores (all >0.90) indicate that the APSQ is “very good” at correctly discriminating moderate, high and very high distress in both male and female athletes [39].

### 2.8. Statistical analysis

Data were split by gender for all analyses. Descriptive statistics were used to describe population data. Participants were categorized according to lumbar BMD z-scores (≤-1 = low BMD, >-1 = normal BMD) [40] and female participants were categorized according to LEAF-Q score (≥8 = at risk, < 8 = low risk) [25].

Variables were assessed for normality using the Shapiro-Wilk test. If data were normally distributed, independent t-tests were used to assess differences between BMD and LEAF-Q groups. Where data were not normally distributed, Mann Whitney U tests were used. Spearman’s-rank correlations (r_s_) were used to explore relationships between weekly training hours and training age, BMD, BMI, body fat%, or sleep duration. Effect sizes were calculated as Hedges’ g and interpreted as small (d = 0.2), medium (d = 0.5), and large (d = 0.8) [41]. Statistical significance was set at *p* < 0.05. All statistical analyses were carried out using Statistics Package for the Social Sciences (SPSS v28, IBM Corp, Armonk, New York).

## 3. Results

Participant demographics are shown in Table 1. Fifty-one percent of participants were triathletes, 20% were cyclists and 29% were runners. Forty-six percent of participants self-reported as recreational athletes, 49% as national age-group athletes and 4% as elite athletes.

**Table 1.** Participant demographics.

	Males		Females	
	Mean	SD	Mean	SD
Age (yrs)	43.7	13.4	45.4	12.5
Height (m)	1.75	0.18	1.64	0.05
Weight (kg)	78.8	9.2	64.4	11.4
Waist Circumference (cm)	82.7	8.4	74.2	8.1
% Body Fat	18.7	4.8	24.0	7.7
Training age (yrs)	11.8	8.1	9.6	8.1
Weekly training time (h)	11.4	4.2	10.2	4.6
Sleep duration (h)	7.3	0.8	6.9	1.0
Perception they fuel well	Yes − 91% No − 9%		Yes − 96% No − 4%	
Restrict dietary intake at times	Yes − 38% No − 62%		Yes − 44% No − 66%	

Male and female BMD results are shown in Table 2. Thirty-one percent of male participants were categorized as having low lumbar BMD. Males with low BMD were significantly older (*p* = 0.031), had been training for a longer period (*p* = 0.005), had a lower maximum adult body weight (*p* = 0.021) and a lower APSQ score (*p* = 0.007) than males with normal BMD.

**Table 2.** Body composition, cardiovascular, metabolic, bone and questionnaire results for male and female participants with normal and low BMD (mean ± SD, hedges’ d).

		Males			Females		
		Normal BMD (*n* = 22)	Low BMD (*n* = 10)	g	Normal BMD (*n* = 21)	Low BMD (*n* = 2)	g
	Age (years)	40 ± 13	52 ± 10*	0.88	46 ± 13	38 ± 9	0.67
	Training age (years)	10 ± 8	16 ± 6*	0.76	10 ± 9	8 ± 5	0.26
Bone Measures	Lumbar T-score	0.55 ± 1.13	− 1.83 ± 0.44*	2.37	0.20 ± 1.01	− 1.45 ± 0.35*	1.60
	Lumbar Z-Score	0.77 ± 1.28	− 1.52 ± 0.38*	2.05	0.93 ± 0.89	− 1.25 ± 0.07*	2.42
	Lumbar BMC (g)	81.77 ± 15.77	71.05 ± 14.19	0.68	65.81 ± 14.43	51.67 ± 12.72	0.95
	Lumbar BMD (g/cm^2^)	1.11 ± 0.16	1.01 ± 0.15	0.69	1.05 ± 0.11	0.89 ± 0.04	1.45
BodyComposition	Body Mass (Kg)	80.3 ± 8.4	75.5 ± 10.5	0.52	64.9 ± 11.8	58.7 ± 1.8	0.52
	BMI (kg/m^2^)	29.4 ± 3.8	24.1 ± 3.1	0.29	24.1 ± 3.8	21.6 ± 2.1	0.64
	WC (cm)	83.0 ± 7.6	81.9 ± 10.5	0.13	74.7 ± 8.2	69.5 ± 4.9	0.62
	Body fat (%)	18.8 ± 4.5	18.5 ± 5.6	0.07	24.4 ± 7.8	19.2 ± 7.1	0.65
	Fat Mass (kg)	19.6 ± 4.8	18.6 ± 5.9	0.19	22.6 ± 8.2	15.7 ± 3.5	0.82
	Fat Free Mass (kg)	59.2 ± 5.0	55.5 ± 5.6	0.69	41.2 ± 4.4	41.9 ± 1.5	0.52
	Visceral Body fat (kg)	0.74 ± 0.31	0.71 ± 0.40	0.14	0.83 ± 0.45	0.41 ± 0.20	0.92
	Highest Adult Body Mass (kg)	91.8 ± 12.4	82.1 ± 11.2*	0.65	74.0 ± 19.4	67.5 ± 10.6	0.33
	Lowest Adult Body Mass (kg)	74.4 ± 8.0	70.7 ± 9.4	0.43	59.8 ± 11.7	51.0 ± 1.4	0.74
Cardiovascular measures	Systolic Pressure (mmHg)	136 ± 13	134 ± 14	0.20	127 ± 15	126 ± 3	0.10
	Diastolic Pressure (mmHg)	83 ± 10	80 ± 7	0.26	78 ± 11	86 ± 1	0.72
	Glucose (Mmol/L)	4.8 ± 0.4	4.8 ± 4	0.17	5.1 ± 0.9	4.9 ± 0.1	0.28
	TC (Mmol/L)	4.6 ± 0.9	4.5 ± 0.6	0.72	4.90 ± 1.17	4.36 ± 0.40	0.46
	TGR Mmol/L)	0.93 ± 0.48	0.87 ± 0.42	0.12	1.33 ± 0.90	0.81 ± 0.16	0.60
	HDL (Mmol/L)	1.58 ± 0.40	1.70 ± 0.33	0.34	1.75 ± 0.65	1.74 ± 0.18	0.02
	LDL (Mmol/L)	2.54 ± 0.74	2.49 ± 0.63	0.06	2.75 ± 0.86	2.25 ± 0.49	0.57
	TC/HDL ratio	3.02 ± 1.03	2.76 ± 0.71	0.27	3.13 ± 0.81	2.55 ± 0.49	0.71
Questionnaires	LEAF-Q				9.00 ± 2.68	8.50 ± 0.71	0.18
	APSQ	17 ± 5	13 ± 5*	0.75	15 ± 3	22 ± 1*	2.06
	ABQ	2.2 ± 0.6	2.1 ± 0.5	0.23	2.2 ± 0.4	3.0 ± 0.8	1.61

*Denotes *p* < 0.05, g*=* effect size, WC= Waist circumference, TC= Total cholesterol, TGR= Triglycerides, HDL= High-density lipoproteins, LDL= Low-density lipoproteins, BMC= Bone mineral content, BMD= Bone mineral density.

Nine percent of females were categorized as having low lumbar BMD. APSQ score was significantly higher in females with low BMD (*p* = 0.016). LEAF-Q score was not significantly different between females with low and normal BMD but both females categorized as low-BMD were classified as at risk of LEA by LEAF-Q score (≥8 points) and reported that their periods had previously stopped for three months or longer.

Female LEAF-Q scores are shown in Table 3. Seventy-seven percent of females were categorized as at risk of LEA. Females at risk of LEA had a significantly higher body weight (*p* = 0.009) and a non-significant trend with a large effect toward a higher BMI and %body fat. Females at risk of LEA had a significantly higher ABQ score (*p* = 0.025) but not ASPQ score. Five females were amenorrheic at the time of the study (periods stopped for 3 months of longer) and these were all categorized as at risk of LEA by LEAF-Q score.

**Table 3.** Body composition, cardiovascular, metabolic, bone and questionnaire results for female participants risk of LEA classified according to LEAF-Q score(mean ± SD, Hedges’ g).

		Low risk (<8 points) n = 5	At Risk (>8 points) n = 17	g
	Age (years)	45 ± 18	45 ± 11	0.05
	Training age (years)	10 ± 9	10 ± 7	0.02
Bone Measures	Lumbar T-score	− 0.60 ± 0.93	0.24 ± 1.10	0.78
	Lumbar Z-Score	0.16 ± 0.43	0.89 ± 1.16	0.69
	Lumbar BMC (g)	58.57 ± 7.94	66.77 ± 16.05	0.55
	Lumbar BMD (g/cm^2^)	1.02 ± 0.06	1.05 ± 0.13	0.20
Body Composition	Body Mass (Kg)	54.7 ± 3.6	66.0 ± 10.9*	1.15
	BMI (kg/m^2^)	21.7 ± 1.8	24.2 ± 3.8	0.70
	WC (cm)	68.8 ± 4.1	75.0 ± 8.0	0.82
	Body fat (%)	19.6 ± 3.8	24.5 ± 8.0	0.67
	Fat Mass (kg)	16.7 ± 1.7	22.7 ± 8.4	0.77
	Fat Free Mass (kg)	37.0 ± 3.1	42.1 ± 3.6	1.43
	Visceral Body fat (kg)	0.53 ± 0.13	0.84 ± 0.47	0.60
	Highest Adult Body Mass (kg)	60.3 ± 7.4	74.1 ± 17.3	0.86
	Lowest Adult Body Mass (kg)	48.8 ± 1.3	60.1 ± 10.7	1.16
Cardiovascular measures	Systolic Pressure (mmHg)	124 ± 11	128 ± 15	0.24
	Diastolic Pressure (mmHg)	75 ± 10	81 ± 10	0.52
	Glucose (Mmol/L)	4.98 ± 0.59	5.12 ± 0.98	0.15
	TC (Mmol/L)	5.26 ± 0.64	4.64 ± 1.18	0.57
	TGR Mmol/L)	1.50 ± 1.75	1.21 ± 0.48	0.31
	HDL (Mmol/L)	2.13 ± 1.13	1.64 ± 0.39	0.79
	LDL (Mmol/L)	3.22 ± 0.60	2.43 ± 0.75*	1.26
	TC/HDL ratio	3.14 ± 0.70	3.00 ± 0.83	0.17
Questionnaires	LEAF-Q	6 ± 2	10 ± 2*	2.42
	APSQ	14 ± 3	16 ± 4	0.40
	ABQ	1.9 ± 0.3	2.4 ± 0.5*	1.10

*Denotes *p* < 0.05, g*=*effect size, WC= Waist circumference, TC= Total cholesterol, TGR= Triglycerides, HDL= High-density lipoproteins, LDL= Low-density lipoproteins, BMC= Bone mineral content, BMD= Bone mineral density.

### 3.1. Training, nutrition and sleep

There were no significant relationships between weekly training hours and training age, BMD, BMI, body fat, or sleep duration (*p* > 0.05). Forty percent of participants reported restricting calorie intake, but 93% reported that they feel that they fuel well. This disparity indicates a possible misunderstanding of the fueling requirements of endurance sport.

## 4. Discussion

The aim of this study was to explore bone health, risk of LEA, and associated factors in endurance athletes. Results showed a high prevalence of low lumbar spine BMD in male athletes, which was associated with older age, longer training history, and poorer psychological status. In contrast, low-BMD was less common in females but the two females with low-BMD reported prior amenorrhea and were classified as at risk of LEA based on LEAF-Q score. Notably, females categorized as at risk of LEA had a higher body weight and body fat percentage than females classified at low risk, which contradicts the common belief that LEA results in weight loss and low body weight.

### 4.1. Bone health

The high proportion of male athletes with low-BMD is concerning and aligns with previous research reporting prevalence’s of 13–50% for osteopenia (Z-score between  − 1 and  − 2) and 0–13% for osteoporosis (Z-score ≤-2) in male endurance athletes [42–45]. In athletes, the most common non-genetic factor for low-BMD is prolonged and/or repeated periods in LEA [6]. The lumbar spine is composed of metabolically active trabecular bone and appears particularly vulnerable to the effects of LEA and associated hormonal disruptions. Our observation that males with low-BMD were older and had longer training histories supports previous findings that prolonged exposure to athletic training may lead to accumulated bone microdamage and suppress bone formation [46]. Low-BMD is a risk factor for fractures and osteoporosis, and increasing the likelihood of fractures in later life, which can cause significant morbidity [47].

Athletes, particularly those in high-impact sports with high mechanical loading, are expected to have a 5%–30% higher BMD than age-matched non-athletes [22], resulting in z-scores above the population norm (i.e. z-score ≥0). However, athletes in sports emphasizing leanness often exhibit lower BMD due to energy deficits from dietary restrictions [42]. Contrary to prior studies indicating that cyclists have lower z-scores due to less load-bearing exercise and a performance-related emphasis on weight, our results showed a higher prevalence of low z-scores in triathletes and runners compared to cyclists (Table 2). This discrepancy might be due to differing performance levels of participants and a relatively low sample size. Additionally, because z-scores are based on general population data, they might not accurately reflect athletes, who typically have a higher BMD. Consequently. the prevalence of low-BMD in this study population may be underestimated [21,48]. Given that low-BMD is two to three times more prevalent in non-athletic premenopausal women than in elite athletes, further research is needed to establish normative BMD values for athletes [17,22].

There was a mean age difference of 12 years between males with normal and low-BMD but no difference in females, possibly due to the low number of females (*n* = 2) with low-BMD in this study. Age-related decline in bone mineral density is primarily caused by reduced calcium supply and absorption, hormonal changes, and decreased levels of physical activity [47]. These causes may differ between males and females, with decreased BMD observed to a greater extent in postmenopausal women due to the significant role of estrogen in maintaining or increasing BMD. Additionally, estrogen levels are associated with factors such as reduced skeletal blood flow, physical inactivity, insufficient calcium intake, decreased gut absorption, reduced hormone function, and genetics [49]. In men, age related BMD loss is attributed to reduced levels of gender hormones, insulin-like growth factor (IGF-1) and mineral-deficient nutrition [49].

A significant finding of the study was that males and females with low-BMD reported higher psychological strain compared to those with normal BMD. This indicates high levels of stress and poorer mental health, suggesting increased psychological strain may accompany physiological energy deficits [2]. Psychological strain is characterized by a combination of perceived stress and difficulty coping, spanning a continuum of emotional exhaustion and reaction to stressful experiences [50]. When coping resources are exceeded, stress-related symptoms of psychological strain may emerge [50]. Psychological stress and/or depression can result from, and contribute to LEA, particularly in individuals susceptible to disordered eating [51]. Although overt eating disorders are less common in male athletes, unintended energy deficits due to poor nutrition knowledge are prevalent and linked to adverse health outcomes [6]. Previous studies have found that athletes with REDs expressed perceived vulnerability and psychological stress related to experiencing inadvertent LEA and/or psychological distress while attempting to maintain optimal EA, with the concept of “long-term recovery” being an ongoing “battle” [52].

### 4.2. LEAF-Q

Results showed that 77% of females were classified as at risk of LEA according to their LEAF-Q score (Table 3). This is slightly higher than previous studies which have reported prevalences of 34–60% of females across varied populations [1,2,25,53]. The LEAF-Q was initially validated in elite athletes [54] and it is possible that it is less sensitive in the more diverse population in this study. Contrary to previous research reporting that females who scored ≥ 8 on the LEAF-Q had lower BMI and body fat [2,12], females at risk of LEA in this study had higher body weight, BMI and body fat percentage. Previous research has shown that athletes with higher average within-day energy deficits have higher body fat percentages than those meeting their energy requirements [55]. Athletes often use energy restriction to achieve desirable body composition, particularly in endurance sports where lighter body weights are believed to enhance performance. Several factors might explain why female athletes at risk of LEA had a higher body weight and body fat percentage. The human adaptive response to energy restriction, including starvation and famine, may result in adaptive thermogenesis, reducing metabolic rate and increasing fat storage from limited energy intake [56,57]. Energy restriction is often linked to carbohydrate restriction, and low carbohydrate diets have been shown to increase fat storage and weight gain over time via metabolic effects involving insulin, leptin, and other hormones [58,59].

Non-elite athletes often consume most of their energy intake outside of exercise periods due to the anorectic effect of exercise and scheduling training around work and family commitments. Failure to meet the energy requirements of training can lead to metabolic and hormonal adaptations and can encourage binge-eating where periods of restrictive eating are followed by excessive eating [60,61]. Additionally, psychological stress can contribute to hypercortisolism, altered secretion of appetite hormones like leptin and ghrelin, and increased fat accumulation [62,63]. Therefore, it is important that athletes seeking to reduce their fat mass or improve their body composition seek advice from sports nutrition professionals to ensure that they reach their goals without compromising their health. Although LEAF-Q thresholds have not been fully validated and may overestimate LEA risk, the high prevalence observed warrants concern and indicates that athletes would benefit from improved nutrition education [64].

### 4.3. Sleep

Participants in this study did not reach the recommended amount of sleep (>8 hours) for adults, or for athletes (9–10 hours) [65]. Studies have found links between disturbed quality sleep and REDs, although the cause and effect relationship is not yet established [66]. Several factors might contribute to low sleep duration, including elevated stress levels, supported by the high levels of psychological strain reported in this study. Individuals might experience insufficient sleep due to stress or heightened stress levels due to inadequate sleep. Studies have shown a high prevalence of sleep disturbances and poor mental health symptoms in Olympic athletes and suggested that LEA in female athletes is highly associated with illness and physiological stress potentially caused by sleep deprivation [15]. Additional research is needed to evaluate these relationships across different sports, competitive levels, and populations.

### 4.4. Cardiovascular measures

Mean blood pressure readings were higher than normal (120/80 mmHg) across all groups within this study [67]. These results contradict previous studies that have found athletes with REDs have low blood pressure [6]. Risk factors in female patients with chronic LEA are similar to those seen in postmenopausal women (i.e. risk for coronary vascular disease increases substantially after menopause), possibly due to the protective role of estrogen for the cardiovascular system. These include unfavorable lipid profiles (elevated levels of LDL, triglycerides, and cholesterol) which put chronically hypoestrogenic female athletes at risk for coronary vascular disease [68]. Studies of LEA in amenorrheic athletes have shown bradycardia and lower blood pressures due to alterations in the renin – angiotensin – aldosterone system [6]. Given the normal blood lipid profiles seen in this athletic population, it is possible that elevated blood pressure might be due to high stress levels. This is supported significantly higher APSQ scores in participants at high risk of REDs or with low lumbar BMD.

Exercise alters the equilibrium of homeostasis in the body and therefore is a form of stress. Once a threshold of stress has been exceeded, the brain evokes the “stress system” along with its peripheral components the hypothalamic-pituitary-adrenal (HPA) axis and the autonomic nervous system [69]. The primary function of the HPA axis is to regulate the stress response. Glucocorticoid hormones are the product of HPA axis activation from the “stress response” and act on multiple bodily systems to maintain homeostasis. The release of cortisol causes several changes that help the body to deal with stress such as increases blood flow, an enhanced ability of the brain to use glucose and increased availability of substances that repair tissues. Cortisol also suppresses functions that would be nonessential or harmful in a fight-or-flight situation [70]. With adequate rest, cortisol levels return to a normal level. However, when cortisol levels remain high due to continuous stress or hypothalamic dysfunction, the secretion of other hormones is affected, causing adverse effects such as amenorrhea, osteoporosis and mental health disorders, similar to those seen in athletes experiencing REDs [71,72]. Results concerning the behavior of cortisol levels are variable in relation to the excessive energy expenditure or physiological and psychological stress in REDs. While some studies show increased basal level of cortisol which is seen in the typical stress response, decreased levels of cortisol are also reported following extreme levels of stress [73–76]. A decrease in cortisol may occur in chronic excessive energy expenditure states like REDs, while an increase might represent acute higher physiological strain. Further investigation is needed to explore cortisol levels interactions in this population and whether the high prevalence of REDs risk is a cause or effect of cortisol level changes [77].

### 4.5. Limitations

This study has several limitations that must be considered. Because the study was cross-sectional, the duration of symptoms experienced by athletes categorized as at risk of LEA is unknown. As a result, it is not possible to determine the time frame over which LEA might develop into REDs. The population in this study was older than in previous research, and some symptoms associated with LEA may be attributable to peri-menopausal changes in females. This could have artificially inflated LEAF-Q scores. Participants were largely middle-aged and all European Caucasians, many of whom began sport as adults and had relatively low training ages. Therefore, physiological symptoms of prolonged LEA (e.g. reduced BMD) might not yet have manifested in females, despite many being identified as at risk of LEA from the LEAF-Q. Additionally, the self-report nature of questionnaires could lead to response bias, with possible over-reporting or under-reporting. Hence, responses must be interpreted with caution.

## 5. Conclusion

There was a high prevalence of low-BMD in male endurance athletes and a high prevalence of female endurance athletes categorized as at risk of LEA. Females at risk of LEA had higher body mass and body fat percentage compared to those at low risk of LEA. Athletes with low-BMD or at risk of LEA exhibited higher psychological strain and burnout scores, suggesting an interaction between psychological stress and nutritional factors. Further research is needed to investigate whether symptoms of LEA are a result of or a cause of high levels of psychological strain. Research is also needed to determine relative contributions of training and nutrition and whether mitigating stress through appropriate interventions might help alleviate some of the negative health and performance consequences.

## Data Availability

Data will be made available by request.

## References

[1] Burke LM, Lundy B, Fahrenholtz IL, et al. Pitfalls of conducting and interpreting estimates of energy availability in free-living athletes. Int J Sport Nutr Exerc Metab. 2018;28:350–16. doi: 10.1123/ijsnem.2018-0142 30029584

[2] Heikura IA, Uusitalo ALT, Stellingwerff T, et al. Low energy availability is difficult to assess but outcomes have large impact on bone injury rates in elite distance athletes. Int J Sport Nutr Exerc Metab. 2018;28(4):403–411. doi: 10.1123/ijsnem.2017-0313 29252050

[3] Stellingwerff T, Heikura IA, Meeusen R, et al. Overtraining syndrome (OTS) and relative energy deficiency in sport (RED-S): shared pathways, symptoms and complexities. Sports Med. 2021;51(11):2251–2280. doi: 10.1007/s40279-021-01491-0 34181189

[4] Wasserfurth P, Palmowski J, Hahn A, et al. Reasons for and consequences of low energy availability in female and male athletes: social environment, adaptations, and prevention. Sports Med - Open. 2020;6(1):44. doi: 10.1186/s40798-020-00275-6 32910256 PMC7483688

[5] Logue D, Madigan SM, Delahunt E, et al. Low energy availability in athletes: a review of prevalence, dietary patterns, physiological health, and sports performance. Sports Med. 2018;48(1):73–96. doi: 10.1007/s40279-017-0790-3 28983802

[6] Mountjoy M, Ackerman KE, Bailey DM, et al. 2023 International Olympic Committee’s (IOC) consensus statement on relative energy deficiency in sport (REDs). Br J Sports Med. 2023;57(17):1073–1098. doi: 10.1136/bjsports-2023-106994 37752011

[7] Kinoshita N, Uchiyama E, Ishikawa-Takata K, et al. Association of energy availability with resting metabolic rates in competitive female teenage runners: a cross-sectional study. J Int Soc Sports Nutr. 2021;18:70. doi: 10.1186/s12970-021-00466-w 34784926 PMC8594218

[8] Wilson G, Martin D, Morton JP, et al. Male flat jockeys do not display deteriorations in bone density or resting metabolic rate in accordance with race riding experience: implications for RED-S. Int J Sport Nutr Exerc Metab. 2018;28:434–439. doi: 10.1123/ijsnem.2017-0371 29431539

[9] Doyle-Lucas AF, Akers JD, Davy BM. Energetic efficiency, menstrual irregularity, and bone mineral density in elite professional female ballet dancers. J Dance Med & Sci. 2010;14(4):146–154. doi: 10.1177/1089313X1001400403 21703085

[10] Loucks AB, Thuma JR. Luteinizing hormone pulsatility is disrupted at a threshold of energy availability in regularly menstruating women. J Clin Endocrinol Metab. 2003;88:297–311. doi: 10.1210/jc.2002-020369 12519869

[11] Redman LM, Loucks AB. Menstrual disorders in athletes. Sports Med. 2005;35:747–755. doi: 10.2165/00007256-200535090-00002 16138785

[12] Papageorgiou M, Elliott-Sale KJ, Parsons A, et al. Effects of reduced energy availability on bone metabolism in women and men. Bone. 2017;105:191–199. doi: 10.1016/j.bone.2017.08.019 28847532

[13] Fagerberg P. Negative consequences of low energy availability in natural male bodybuilding: a review. 2018 [cited 2024 Jun 5]. doi: 10.1123/ijsnem.2016-0332 28530498

[14] Melin AK, Heikura IA, Tenforde A, et al. Energy availability in athletics: health, performance, and physique. Int J Sport Nutr Exerc Metab. 2019;29:152–164. doi: 10.1123/ijsnem.2018-0201 30632422

[15] Drew M, Vlahovich N, Hughes D, et al. Prevalence of illness, poor mental health and sleep quality and low energy availability prior to the 2016 Summer Olympic games. Br J Sports Med. 2018;52(1):47–53. doi: 10.1136/bjsports-2017-098208 29056598

[16] Langbein RK, Martin D, Allen-Collinson J, et al. “It’s hard to find balance when you’re broken”: exploring female endurance athletes’ psychological experience of recovery from relative energy deficiency in sport (RED-S). Perform Enhancement Health. 2022;10:100214. doi: 10.1016/j.peh.2021.100214

[17] Tenforde AS, Fredericson M. Influence of sports participation on bone health in the young athlete: a review of the literature. Pm R. 2011;3:861–867. doi: 10.1016/j.pmrj.2011.05.019 21944303

[18] Hind K, Truscott JG, Evans JA. Low lumbar spine bone mineral density in both male and female endurance runners. Bone. 2006;39(4):880–885. doi: 10.1016/j.bone.2006.03.012 16682267

[19] Bennell KL, Malcolm SA, Khan KM, et al. Bone mass and bone turnover in power athletes, endurance athletes, and controls: a 12-month longitudinal study. Bone. 1997;20(5):477–484. doi: 10.1016/s8756-3282(97)00026-4 9145246

[20] Barrack MT, Gibbs JC, De Souza MJ, et al. Higher incidence of bone stress injuries with increasing female Athlete triad–related risk factors: a prospective multisite study of exercising girls and women. Am J Sports Med. 2014;42(4):949–958. doi: 10.1177/0363546513520295 24567250

[21] Jonvik KL, Torstveit MK, Sundgot-Borgen J, et al. Do we need to change the guideline values for determining low bone mineral density in athletes? J Appl Physiol (1985). 2022;132(5):1320–1322. doi: 10.1152/japplphysiol.00851.2021 35060767 PMC9126212

[22] Torstveit MK, Sundgot-Borgen J. Low bone mineral density is two to three times more prevalent in non-athletic premenopausal women than in elite athletes: a comprehensive controlled study. Br J Sports Med. 2005;39(5):282–287. doi: 10.1136/bjsm.2004.012781 15849292 PMC1725217

[23] van Staa TP, Dennison EM, Leufkens HG, et al. Epidemiology of fractures in England and Wales. Bone. 2001;29(6):517–522. doi: 10.1016/s8756-3282(01)00614-7 11728921

[24] Burke LM, Cox GR, Cummings NK, et al. Guidelines for daily carbohydrate intake: do athletes achieve them? Sports Med. 2001;31(4):267–299. doi: 10.2165/00007256-200131040-00003 11310548

[25] Melin A, Tornberg ÅB, Skouby S, et al. The LEAF questionnaire: a screening tool for the identification of female athletes at risk for the female athlete triad. Br J Sports Med. 2014;48(7):540–545. doi: 10.1136/bjsports-2013-093240 24563388

[26] Lundy B, Torstveit MK, Stenqvist TB, et al. Screening for low energy availability in male athletes: attempted validation of LEAM-Q. Nutrients. 2022;14(9):1873. doi: 10.3390/nu14091873 35565840 PMC9101736

[27] Beals KA, Hill AK. The prevalence of disordered eating, menstrual dysfunction, and low bone mineral density among US collegiate athletes. Int J Sport Nutr Exerc Metab. 2006;16:1–23. doi: 10.1123/ijsnem.16.1.1 16676700

[28] Kuikman MA, Mountjoy M, Burr JF. Examining the relationship between exercise dependence, disordered eating, and low energy availability. Nutrients. 2021;13(8):2601. doi: 10.3390/nu13082601 34444761 PMC8398044

[29] De Francisco C, Arce C, Del Vílchez MP, et al. Antecedents and consequences of burnout in athletes: perceived stress and depression. Int J Clin Health Psychol. 2016;16(3):239–246. doi: 10.1016/j.ijchp.2016.04.001 30487867 PMC6225055

[30] Eklund RC, DeFreese JD. Athlete burnout. Handbook of sport psychology [internet]. John Wiley & Sons, Ltd; 2020 [cited 2024 Jun 5]. Available from: https://onlinelibrary.wiley.com/doi/abs/10.1002/9781119568124.ch60

[31] Carey M, Markham C, Gaffney P, et al. Validation of a point of care lipid analyser using a hospital based reference laboratory. Ir J Med Sci. 2006;175(4):30–35. doi: 10.1007/BF03167964 Cited: in: PMID: 17312826. 17312826

[32] Bilsborough JC, Greenway K, Opar D, et al. The accuracy and precision of DXA for assessing body composition in team sport athletes. J Sports Sci. 2014;32(19):1821–1828. doi: 10.1080/02640414.2014.926380 Cited: in: PMID: 24914773. 24914773

[33] Barrack MT, Fredericson M, Tenforde AS, et al. Evidence of a cumulative effect for risk factors predicting low bone mass among male adolescent athletes. Br J Sports Med. 2017;51(3):200–205. doi: 10.1136/bjsports-2016-096698 Cited: in: PMID: 29461218. 29461218

[34] Gerber M, Gustafsson H, Seelig H, et al. Usefulness of the Athlete burnout questionnaire (ABQ) as a screening tool for the detection of clinically relevant burnout symptoms among young elite athletes. Psychol Sport Exercise. 2018;39:104–113. doi: 10.1016/j.psychsport.2018.08.005

[35] Raedeke TD, Smith AL. Development and preliminary validation of an athlete burnout measure. 2001 [cited 2024 Jun 6]. doi: 10.1123/jsep.23.4.281 28682196

[36] Cresswell SL, Eklund RC. Motivation and burnout among top amateur rugby players. Med Sci Sports Exerc. 2005;37:469–477. doi: 10.1249/01.mss.0000155398.71387.c2 Cited: in: PMID: 15741847. 15741847

[37] Lonsdale C, Hodge K, Rose E. Athlete burnout in elite sport: a self-determination perspective. J Sports Sci. 2009;27:785–795. doi: 10.1080/02640410902929366 Cited: in: PMID: 19437185. 19437185

[38] Rice SM, Parker AG, Mawren D, et al. Preliminary psychometric validation of a brief screening tool for athlete mental health among male elite athletes: the Athlete psychological strain questionnaire. Int J Sport Exercise Phychol. 2020;18(6):850–865. doi: 10.1080/1612197X.2019.1611900

[39] Rice S, Olive L, Gouttebarge V, et al. Mental health screening: severity and cut-off point sensitivity of the Athlete psychological strain questionnaire in male and female elite athletes. BMJ Open Sport Exerc Med. 2020;6(1):e000712. doi: 10.1136/bmjsem-2019-000712 Cited: in: PMID: 32231792. PMC7101042 32231792

[40] Nattiv A, Loucks AB, Manore MM, et al. American college of sports medicine. American college of sports medicine position stand. The female athlete triad. Med Sci Sports Exerc. 2007;39:1867–1882. doi: 10.1249/mss.0b013e318149f111 Cited: in: PMID: 17909417. 17909417

[41] Hedges LV. Distribution theory for Glass’s estimator of effect size and related estimators. J Educ Stat. 1981;6:107–128. doi: 10.2307/1164588

[42] Andersen OK, Clarsen B, Garthe I, et al. Bone health in elite Norwegian endurance cyclists and runners: a cross-sectional study. BMJ Open Sport Exercise Med. 2018;4(1):e000449. doi: 10.1136/bmjsem-2018-000449 PMC6326301 30687513

[43] Keay N, Francis G, Hind K. Low energy availability assessed by a sport-specific questionnaire and clinical interview indicative of bone health, endocrine profile and cycling performance in competitive male cyclists. BMJ Open Sport Exerc Med. 2018;4(1):e000424. doi: 10.1136/bmjsem-2018-000424 Cited: in: PMID: 30364549. PMC6196965 30364549

[44] Rector RS, Rogers R, Ruebel M, et al. Participation in road cycling vs running is associated with lower bone mineral density in men. Metabolism. 2008;57(2):226–232. doi: 10.1016/j.metabol.2007.09.005 Cited: in: PMID: 18191053. 18191053

[45] Stewart AD, Hannan J. Total and regional bone density in male runners, cyclists, and controls. Med Sci Sports Exerc. 2000;32:1373–1377. doi: 10.1097/00005768-200008000-00003 Cited: in: PMID: 10949001. 10949001

[46] Hutson MJ, O’Donnell E, Brooke-Wavell K, et al. Effects of low energy availability on bone health in endurance athletes and high-impact exercise as a potential countermeasure: a narrative review. Sports Med. 2021;51(3):391–403. doi: 10.1007/s40279-020-01396-4 Cited: in: PMID: 33346900. 33346900 PMC7900047

[47] Gregson CL, Armstrong DJ, Bowden J, et al. UK clinical guideline for the prevention and treatment of osteoporosis. Arch Osteoporos. 2022;17(1):58. doi: 10.1007/s11657-022-01061-5 Cited: in: PMID: 35378630. 35378630 PMC8979902

[48] De Souza MJ, Williams NI, Nattiv A, et al. Misunderstanding the female athlete triad: refuting the IOC consensus statement on relative energy deficiency in sport (RED-S). Br J Sports Med. 2014;48(20):1461–1465. doi: 10.1136/bjsports-2014-093958 Cited: in: PMID: 25037200. 25037200

[49] Kopiczko A, Adamczyk JG, Gryko K, et al. Bone mineral density in elite masters athletes: the effect of body composition and long-term exercise. Eur Rev Aging Phys Act. 2021;18(1):7. doi: 10.1186/s11556-021-00262-0 34058982 PMC8166030

[50] Boswell WR, Olson-Buchanan JB, LePine MA. Relations between stress and work outcomes: the role of felt challenge, job control, and psychological strain. J Vocational Behav. 2004;64(1):165–181. doi: 10.1016/S0001-8791(03)00049-6

[51] Stice E, South K, Shaw H. Future directions in Etiologic, prevention, and treatment research for eating disorders. J Clin Child Adolesc Phychol. 2012;41:845–855. doi: 10.1080/15374416.2012.728156 Cited: in: PMID: 23057638. 23057638

[52] Langbein RK, Martin D, Allen-Collinson J, et al. “I’d got self-destruction down to a fine art”: a qualitative exploration of relative energy deficiency in sport (RED-S) in endurance athletes. J Sports Sci. 2021;39:1555–1564. doi: 10.1080/02640414.2021.1883312 Cited: in: PMID: 33573478. 33573478

[53] Kraus E, Tenforde AS, Nattiv A, et al. Bone stress injuries in male distance runners: higher modified female athlete triad cumulative risk assessment scores predict increased rates of injury. Br J Sports Med. 2019;53(4):237–242. doi: 10.1136/bjsports-2018-099861 Cited: in: PMID: 30580252. 30580252

[54] Melin A, Tornberg AB, Skouby S, et al. The LEAF questionnaire: a screening tool for the identification of female athletes at risk for the female athlete triad. Br J Sports Med. 2014;48(7):540–545. doi: 10.1136/bjsports-2013-093240 Cited: in: PMID: 24563388. 24563388

[55] Deutz RC, Benardot D, Martin DE, et al. Relationship between energy deficits and body composition in elite female gymnasts and runners. Med Sci Sports Exerc. 2000;32:659–668. doi: 10.1097/00005768-200003000-00017 Cited: in: PMID: 10731010. 10731010

[56] Rosenbaum M, Leibel RL. Adaptive thermogenesis in humans. Int J Obes. 2010;34(S1):S47–S55. doi: 10.1038/ijo.2010.184 PMC3673773 20935667

[57] Trexler ET, Smith-Ryan AE, Norton LE. Metabolic adaptation to weight loss: implications for the athlete. J Int Soc Sports Nutr. 2014;11(1):7. doi: 10.1186/1550-2783-11-7 24571926 PMC3943438

[58] Loucks AB, Heath EM. Induction of low-T3 syndrome in exercising women occurs at a threshold of energy availability. Am J Physiol. 1994;266(3):R817–R823. doi: 10.1152/ajpregu.1994.266.3.R817 Cited: in: PMID: 8160876. 8160876

[59] Volek JS, Sharman MJ, Love DM, et al. Body composition and hormonal responses to a carbohydrate-restricted diet. Metabolism. 2002;51(7):864–870. doi: 10.1053/meta.2002.32037 Cited: in: PMID: 12077732. 12077732

[60] Doucet E, Imbeault P, St-Pierre S, et al. Appetite after weight loss by energy restriction and a low-fat diet–exercise follow-up. Int J Obes Relat Metab Disord. 2000;24(7):906–914. doi: 10.1038/sj.ijo.0801251 Cited: in: PMID: 10918539. 10918539

[61] Thaler JP, Yi C-X, Schur EA, et al. Obesity is associated with hypothalamic injury in rodents and humans. J Clin Invest. 2012;122(1):153–162. doi: 10.1172/JCI59660 Cited: in: PMID: 22201683. 22201683 PMC3248304

[62] McLean JA, Barr SI, Prior JC. Cognitive dietary restraint is associated with higher urinary cortisol excretion in healthy premenopausal women. Am J Clin Nutr. 2001;73:7–12. doi: 10.1093/ajcn/73.1.7 Cited: in: PMID: 11124742. 11124742

[63] Tomiyama AJ, Mann T, Vinas D, et al. Low calorie dieting increases cortisol. Psychosom Med. 2010;72:357–364. doi: 10.1097/PSY.0b013e3181d9523c Cited: in: PMID: 20368473. 20368473 PMC2895000

[64] Mathisen TF, Sundgot-Borgen C, Anstensrud B, et al. Intervention in professional dance students to increase mental health- and nutrition literacy: a controlled trial with follow up. Front Sports Act Living. 2022;4. doi: 10.3389/fspor.2022.727048 PMC9532567 36213449

[65] Singh M, Bird SP, Charest J, et al. Sleep and athletes. Operative techniques in sports medicine. 2022;30(1):150897. doi: 10.1016/j.otsm.2022.150897

[66] Kalpana K, Cherian KS, Khanna GL. Energy availability and RED-S risk assessment among kho-kho players in India. Sport Sci Health. 2022:1–8. doi: 10.1007/s11332-022-00996-z Cited: in: PMID: 36061453. PMC9425793 36061453

[67] NICE. Hypertension in adults: diagnosis and management [internet]. NICE; 2019 [cited 2024 Jun 5]. Available from: https://www.nice.org.uk/guidance/ng136

[68] Grosman-Rimon L, Wright E, Freedman D, et al. Can improvement in hormonal and energy balance reverse cardiovascular risk factors in athletes with amenorrhea? Am J Physiol Heart Circ Physiol. 2019;317:H487–H495. doi: 10.1152/ajpheart.00242.2019 Cited: in: PMID: 31322425. 31322425

[69] Mastorakos G, Pavlatou M, Diamanti-Kandarakis E, et al. Exercise and the stress system. Hormones (Athens). 2005;4(2):73–89. Cited: in: PMID: 16613809. 16613809

[70] Smith SM, Vale WW. The role of the hypothalamic-pituitary-adrenal axis in neuroendocrine responses to stress. Dialogues Clin Neurosci. 2006;8(4):383–395. doi: 10.31887/DCNS.2006.8.4/ssmith 17290797 PMC3181830

[71] Tasker JG, Herman JP. Mechanisms of rapid glucocorticoid feedback inhibition of the hypothalamic–pituitary–adrenal axis. Stress. 2011;14(4):398–406. doi: 10.3109/10253890.2011.586446 21663538 PMC4675656

[72] Vedder H. Physiology of the hypothalamic–pituitary–adrenocortical axis. NeuroImmune biology. Elsevier; 2007 [cited 2023 Sep 7]. Available from: https://www.sciencedirect.com/science/article/pii/S1567744307002025

[73] Adlercreutz H, Härkönen M, Kuoppasalmi K, et al. Effect of training on plasma anabolic and catabolic steroid hormones and their response during physical exercise. Int J Sports Med. 1986;7(Suppl 1):S27–S28. doi: 10.1055/s-2008-1025798 3744643

[74] Barron JL, Noakes TD, Levy W, et al. Hypothalamic dysfunction in overtrained athletes. J Clin Endocrinol Metab. 1985;60:803–806. doi: 10.1210/jcem-60-4-803 Cited: in: PMID: 2982908. 2982908

[75] Lehmann M, Gastmann U, Petersen KG, et al. Training-overtraining: performance, and hormone levels, after a defined increase in training volume versus intensity in experienced middle- and long-distance runners. Br J Sports Med. 1992;26(4):233–242. doi: 10.1136/bjsm.26.4.233 Cited: in: PMID: 1490214. 1490214 PMC1479002

[76] Meeusen R, Piacentini MF, Busschaert B, et al. Hormonal responses in athletes: the use of a two bout exercise protocol to detect subtle differences in (over) training status. Eur J Appl Physiol. 2004;91(2–3):140–146. doi: 10.1007/s00421-003-0940-1 14523562

[77] Lehmann M, Foster C, Dickhuth HH, et al. Autonomic imbalance hypothesis and overtraining syndrome. Med Sci Sports Exerc. 1998;30:1140–1145. doi: 10.1097/00005768-199807000-00019 9662686

